# Primary Hepatic Neuroendocrine Tumor with Unusual Thyroid Follicular-Like Morphologic Characteristics

**DOI:** 10.1155/2017/7931975

**Published:** 2017-02-20

**Authors:** Mohd Elmugtaba Ibrahim, Kerolos Abadeer, Qihui (Jim) Zhai, Aziza Nassar

**Affiliations:** ^1^Division of Neurology, Massey Cancer Center, VCU Medical Center, Richmond, VA, USA; ^2^Division of Nephrology and Hypertension, Mayo Clinic, Jacksonville, FL, USA; ^3^Department of Laboratory Medicine and Pathology, Mayo Clinic, Jacksonville, FL, USA

## Abstract

We describe a primary hepatic neuroendocrine tumor of a 57-year-old Thai woman who presented in 2004 with a suspicious mass in the left hepatic lobe. She underwent left hepatectomy for the 10.5-cm mass, called* intermediate grade neuroendocrine carcinoma of unknown origin*,* likely metastatic*. The tumor recurred in 2007, then called* recurrent primary hepatic neuroendocrine tumor* (PHNET), and the patient underwent liver transplant. Because of similarity between the neuroendocrine tumor and a thyroid tumor—specifically, follicular-like characteristics—immunohistochemical stains for thyroglobulin, TTF1, and calcitonin were performed. However, all were negative. All imaging studies revealed no evidence of a primary lesion other than the liver mass. In 2008, the patient's liver transplant failed because of ischemic cholangiopathy, and she underwent a second liver transplant. Seven years later, in 2015, she presented with metastatic neuroendocrine tumor of intermediate grade to the lung, consistent with metastatic PHNET. She underwent left upper-lobe wedge resection to remove the tumor. The patient is alive with no evidence of disease at 13 years after initial diagnosis. This rare variant of PHNET had thyroid-like morphologic characteristics but there is no evidence of primary thyroid tumor or thyroid markers in the primary and recurrent hepatic tumors and lung metastasis.

## 1. Introduction

Primary hepatic neuroendocrine tumors (PHNETs) are rare and poorly described entities, with fewer than 150 cases described in the literature [1]. First described by Edmonson [2] in 1958, PHNETs represent about 0.3% of all neuroendocrine tumors. Therefore, identification and description of these tumors are important to help better understand the clinicopathologic features and biologic behaviors. Moreover, even fewer literature reports deal with the progression and prognosis of PHNETs, which make this case, with a follow-up of 13 years, a distinctive and interesting report.

Neuroendocrine tumors (NETs) in general are relatively rare tumors with an incidence rate of 2 per 100,000 cases of all gastrointestinal tract tumors [3]. NETs can arise virtually anywhere in the body, and approximately 70% of these rare tumors arise in the gastrointestinal tract and lungs [4]; the liver is the common site for metastasis, yet it is an uncommon site for tumor origin.

Our review showed that demographically most of patients with PHNETs are of Asian descent (mean [range] age, 45 [8–87] years) [3, 5]. There is no sex predilection, but a predominance of female patients has been reported (female to male ratio, 1.4 : 1) [2]. In the few reported cases of PHNET and for the present patient, a palpable abdominal mass or abdomen discomfort may be the only presenting concern [1]. This presentation is in contrast to the classic symptoms of carcinoid syndrome that accompany 10% of all NET liver metastasis but rarely occur in PHNET. On imaging, PHNETs tend to appear more vascular than metastatic because of a rich vascular supply from the hepatic artery [6], consistent with the magnetic resonance imaging (MRI) findings of the recurrent PHNET in this case.

## 2. Case Presentation

The patient was a 57-year-old Thai woman who was referred in August 2004 for a suspicious mass in the left hepatic lobe that had been discovered on ultrasonography. She underwent a left hepatectomy of the native liver for an estimated 10-cm mass. Grossly, the resected lobe showed both a 10.5 × 10.0 × 7.0 cm, tannish yellow, focally hemorrhagic, well-circumscribed mass and a 0.7-cm well-circumscribed yellow nodule that were within 3.5 cm of each other.

**Figure 1:**
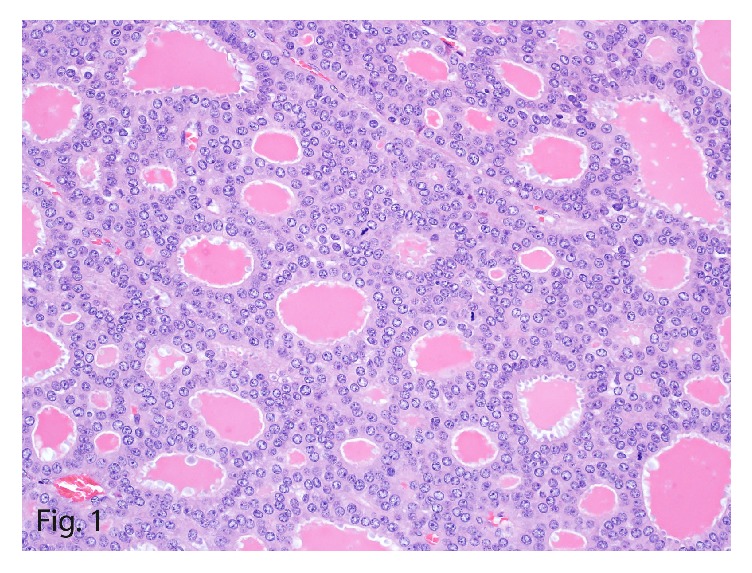
Hepatic tumor with multiple acinar-like structures. These structures contain eosinophilic secretions (thyroid-like morphologic characteristic) with brisk mitotic figures and neoplastic cells with “salt and pepper” chromatin (hematoxylin-eosin, original magnification ×40).

**Figure 2:**
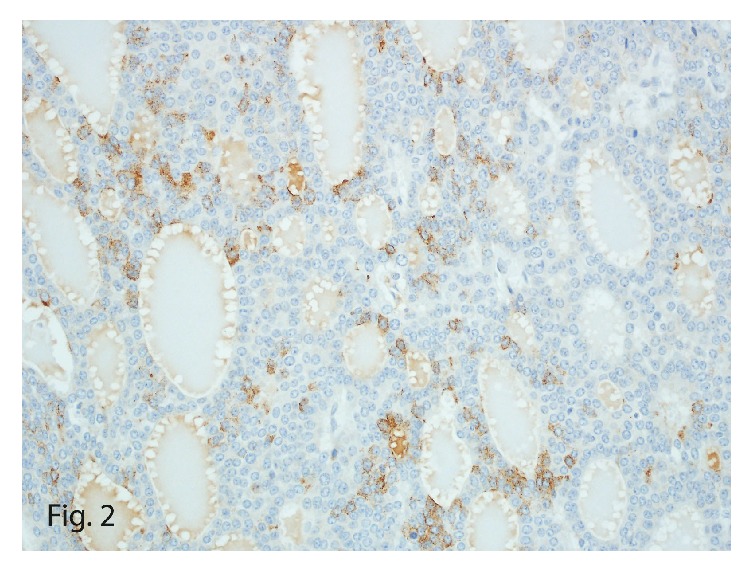
Chromogranin immunostain with focal expression in the tumor (original magnification ×40).

**Figure 3:**
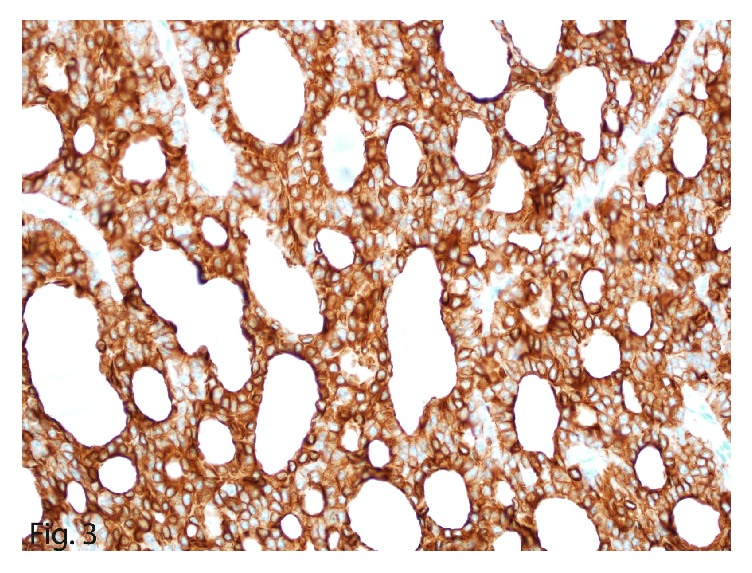
Cytokeratin 7 immunostain in the neoplastic cells (original magnification ×40).

**Figure 4:**
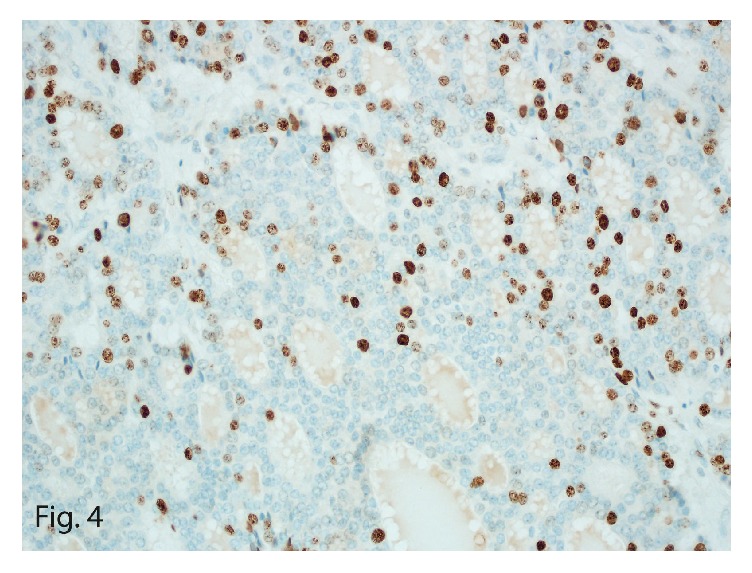
Ki-67 expression in tumor cells (20% mitotic labeling index, original magnification ×40).

On histomorphologic evaluation, the mass showed a monotonous uniform population of neoplastic cells arranged in follicles with intraluminal eosinophilic secretions. The cells were round to oval with scant eosinophilic cytoplasm that had “salt-and-pepper” chromatin (Figure 1) and brisk mitotic activity. Immunohistochemical staining of the larger mass showed focal reactivity for chromogranin (Figure 2) and diffuse reactivity for synaptophysin, consistent with the diagnosis of a neuroendocrine carcinoma. The tumor showed diffuse positive immunostain for keratin and cytokeratin 7 (Figure 3) and patchy reactivity with vimentin. Additional immunostains, including thyroglobulin, thyroid transcription factor (TTF1), and neuron-specific enolase, were negative, as was Congo red, a stain particularly for amyloid. Polyclonal carcinoembryonic antigen immunostain was also negative for the canalicular pattern usually seen in hepatocellular carcinoma, making the diagnosis unlikely. With a mitotic count of 16 or 17 mitoses per 10 high-power fields (HPFs) and Ki-67 labeling index of 20% (Figure 4), the diagnosis of intermediate grade (G2) NET, likely metastatic, was made.

**Figure 5:**
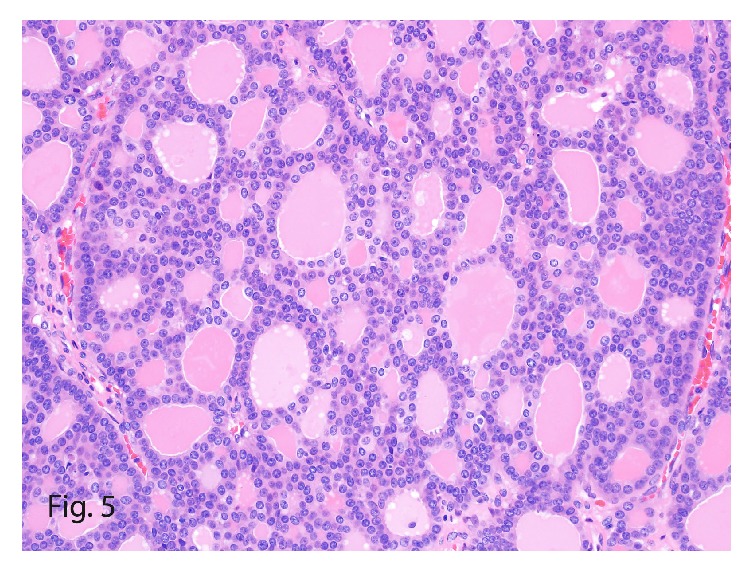
Recurrent hepatic neuroendocrine tumor in 2007 with low-grade morphologic characteristics and a low mitotic count (hematoxylin-eosin, original magnification ×40).

A follow-up MRI in 2007 revealed an 8.9-cm liver tumor on segment VIII, described as circumscribed and hypervascular. No other primary lesion was detected on other imaging, including octreoscan, bone scan, ultrasonography of both breasts and thyroid gland, and endoscopy including a capsule study. Subsequently, the patient underwent a liver transplant following hepatectomy of the remaining native liver. The completion hepatectomy specimen of the remaining native liver showed that the liver capsule was brownish tan, focally fibrotic, and diffusely covered with adhesions. Sectioning revealed an orange well-circumscribed mass measuring 14.5 × 13.0 × 12.1 cm. The tumor had areas of cystic degeneration intermixed with areas of hemorrhage, with a variegated appearance. On microscopy, the tumor showed follicular thyroid-like morphologic characteristics reminiscent of a thyroid follicular carcinoma (Figure 5). However, no immunohistochemical evidence of thyroid differentiation was found, as shown by negative immunoreactivity to thyroglobulin, TTF1, and calcitonin.

**Figure 6:**
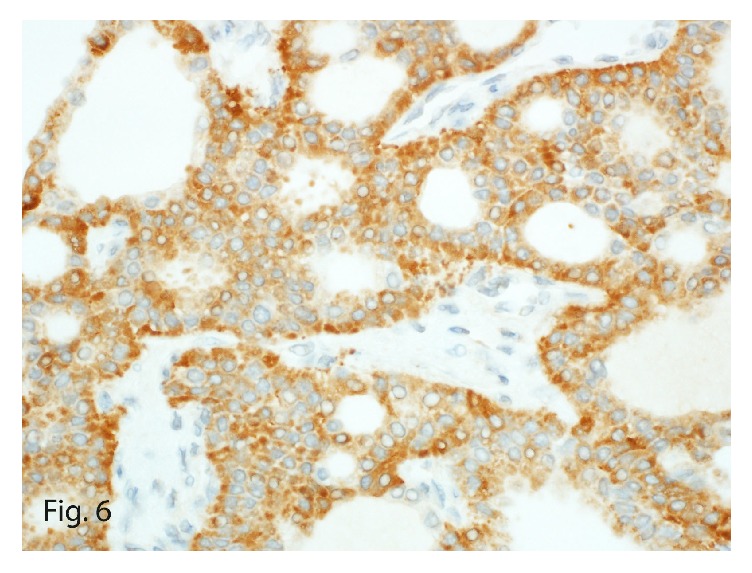
Synaptophysin immunoexpression in the tumor (original magnification ×40).

**Figure 7:**
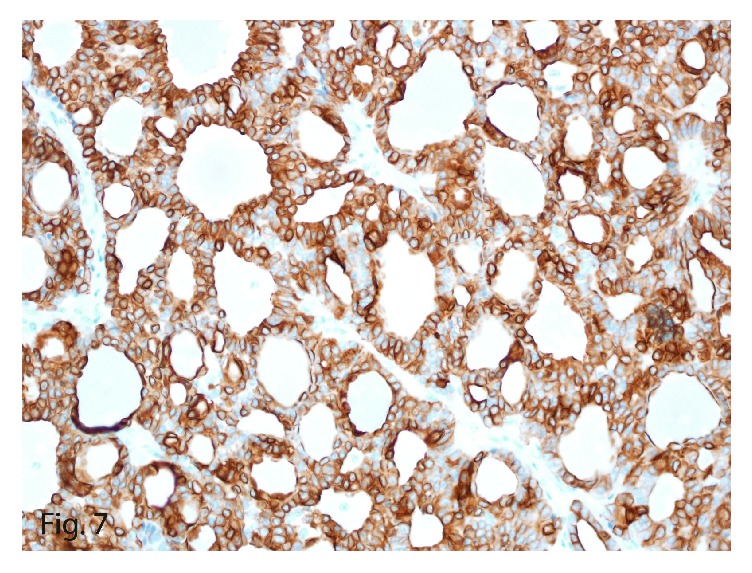
Cytokeratin 7 immunoexpression in the neoplastic cells (original magnification ×40).

**Figure 8:**
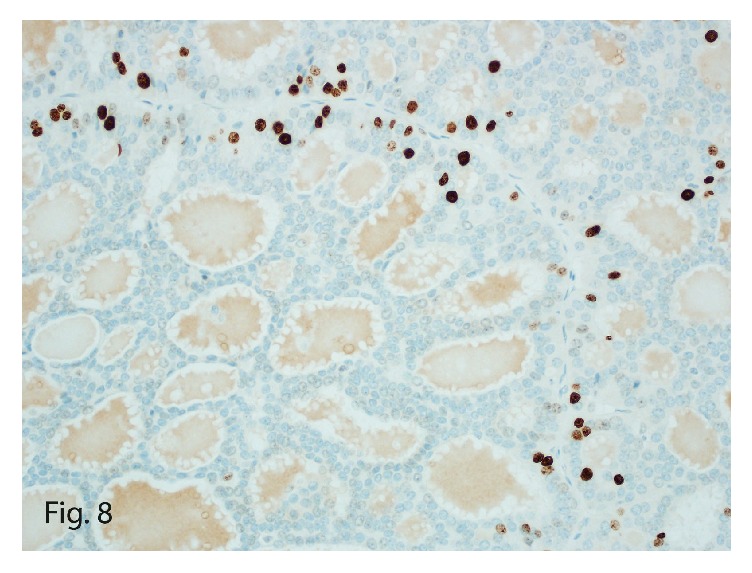
Ki-67 expression in the tumor cells (2% mitotic labeling index, original magnification ×40).

The tumor once again stained positive for neuroendocrine markers, including synaptophysin (Figure 6) and chromogranin, in addition to keratin, cytokeratin 7 (Figure 7), and vimentin, confirming the diagnosis of recurrent PHNET. The lack of the carcinoid symptoms that usually accompany metastatic hepatic NETs and the failure to identify a primary tumor elsewhere on complete body imaging led to the diagnosis of recurrent PHNET. Interestingly, this tumor was a low-grade (G1) tumor with a mitotic count of 1 per 10 HPFs and a Ki-67 labeling index of 2% (Figure 8).

In 2008, the patient's transplanted liver failed because of ischemic cholangiopathy, and she underwent a second liver transplant. On a posttransplant follow-up visit in 2015, a radiograph showed an enlarged left upper-lobe lung nodule. However, an octreoscan and a positron emission tomography/computed tomography scan for whole-body imaging were negative. The nodule size was found to be increasing on interval monitoring; in 2016, the patient underwent a wedge resection. Ultrasonography and MRI of the abdomen showed no liver abnormalities this time.

**Figure 9:**
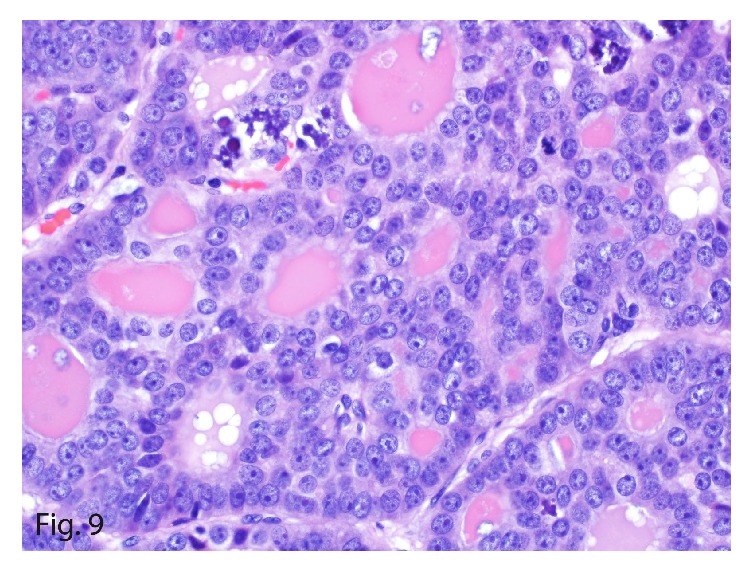
Metastatic hepatic neuroendocrine tumor in 2016 with morphologic characteristics similar to the previous hepatic tumors and an intermediate mitotic count (hematoxylin-eosin, original magnification ×40).

**Figure 10:**
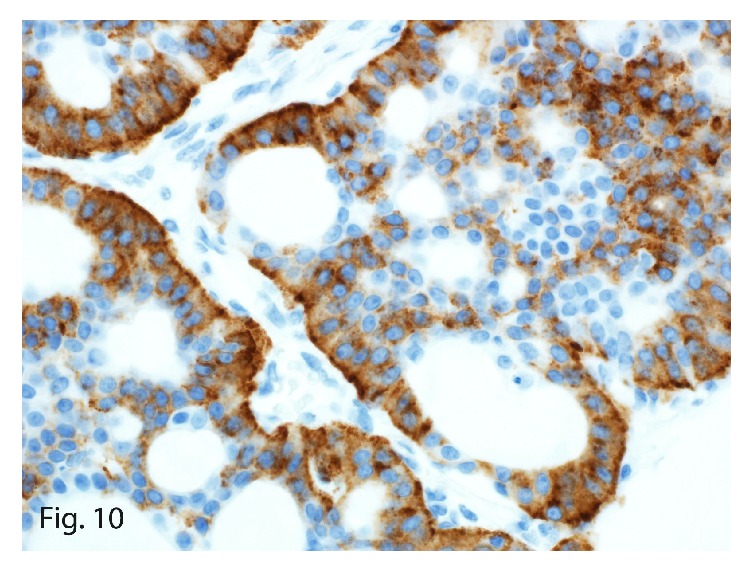
Synaptophysin immunoexpression in the tumor (original magnification ×40).

**Figure 11:**
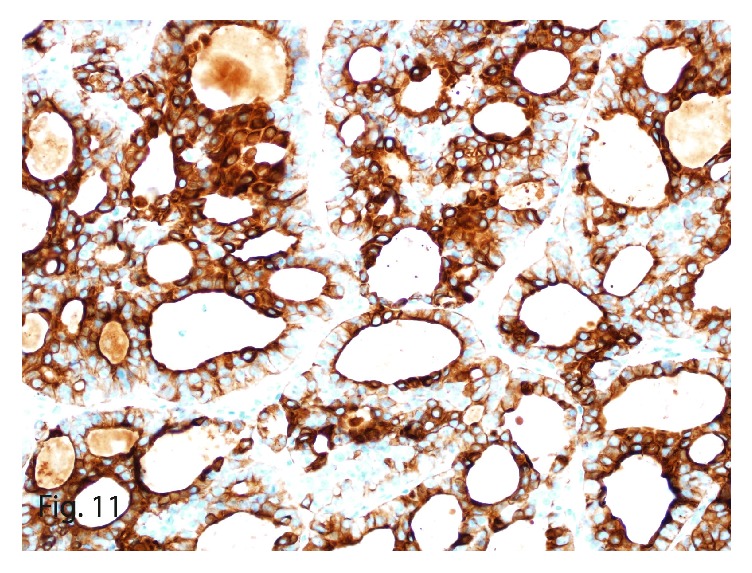
Cytokeratin 7 immunostain with strong and diffuse expression in the neoplastic cells (original magnification ×40).

**Figure 12:**
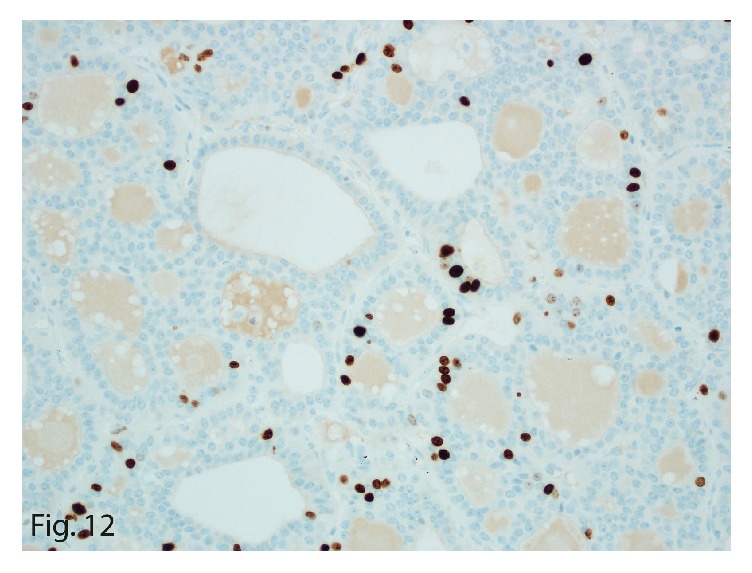
Ki-67 expression in the tumor cells. The mitotic labeling index was 5% (original magnification ×40).

The left upper-lobe lung wedge resection of the suspicious nodule showed a 1.4 × 1.0 × 0.6 cm, well-circumscribed, tan firm mass. On microscopy, the tumor showed similar morphologic characteristics to the 2 previous primary and recurrent hepatic tumors (Figure 9). Immunohistochemically, it stained positive for synaptophysin (focally; Figure 10) and cytokeratin 7 (Figure 11) and was negative for thyroglobulin, chromogranin, PAX8, cytokeratin 20, TTF1, mammaglobin, napsin A, estrogen receptor, GATA3, and c-kit. The tumor characteristics and immunohistochemical profile were consistent with the diagnosis of PHNET metastasis. The Ki-67 labeling index was 5% (Figure 12) with a mitotic count of 2 mitoses per 10 HPFs, thereby grading the metastasis as G2.

## 3. Discussion

PHNET primarily occurs in patients aged 40 to 50 years and is usually located in the right lobe of the liver [3]. PHNETs tend to be large and well-circumscribed, often arising on the right lobe of the liver and surrounded by healthy parenchyma in most cases, with cirrhosis found only as the concomitant liver disease [5, 7–9]. These findings are similar to our case of PHNET except for the PHNET location on the left lobe. However, varying microscopic morphologic characteristics are a commonly described feature of PHNETs. Our case revealed a thyroid follicular-like morphologic appearance reminiscent of thyroid follicular carcinoma, a feature that has never been described previously, to our knowledge. One case showed signet ring cells that were mucin negative [10], but cytokeratin positivity and other various morphologic traits have also been described [9–11].

The origin of PHNET is still unknown, but possible and plausible theories include transformation of liver stem cells; neuroendocrine differentiation of ectopic adrenal tissue or of heterotopic pancreatic tissue in the liver; and transformation of neuroendocrine cells of the intrahepatic biliary ductal epithelium [2].

Immunohistochemistry is by far the most important diagnostic tool with regard to NETs and PHNETs. NETs almost always stain positively for neuroendocrine markers (synaptophysin and, frequently, chromogranin A). Synaptophysin is a peptide of small synaptic vesicles present in all neuroendocrine cells, and thus it is almost always positive, independent of the number of vesicles. Chromogranin A, by comparison, is part of the neurosecretory hormone granules; therefore, evidence of its presence is dependent on the number of vesicles present. Immunohistochemical staining is also useful in differentiating between NETs from other sites of origin and PHNET. TTF1 stain is frequently positive for NETs that originate from the lung, and neuron-specific enolase stain is positive in some NETs of gastroenteropancreatic origin. Both stains tested negative in this particular PHNET case. Other immunostains could be used to differentiate between a liver metastasis and PHNET. For example, thyroglobulin and calcitonin were especially useful in this case to rule out thyroid origin, which was suspected because of the thyroid-like follicular morphologic pattern.

Recently, a rare variant of cholangiocarcinoma with thyroid-like morphologic traits has been described in 2 case reports [12, 13]. However, unlike our case, the newly described cholangiocarcinoma in both case reports did not show neuroendocrine expression, and 1 patient died within 18 months postoperatively.

The present hepatic NET is unusual in its immunohistochemical profile, since all tumors (primary and recurrent hepatic tumors as well as metastasis) expressed cytokeratin 7, which is known to be expressed in cholangiocarcinoma. In addition, cholangiocarcinoma can have neuroendocrine differentiating traits, but they are usually 2 components—a conventional cholangiocarcinoma and a separate component with neuroendocrine differentiation are usually present [14]. Neuroendocrine cells are frequently found in the bile ducts and, therefore, could be expected in cholangiocarcinoma. However, the present case expressed neuroendocrine markers (synaptophysin and chromogranin) in the entire tumor, which showed a thyroid follicular-like morphologic characteristic. Furthermore, cholangiocarcinoma has a shorter, 5-year survival rate of 32% following operative intervention [15], and our patient survived for 13 years after operation.

NETs are classified as well differentiated (tumors) and poorly differentiated (carcinomas). They are graded into G1 and G2 tumors and high-grade (G3) carcinomas on the basis of their mitotic count or the Ki-67 labeling index, or both, with the latter being more accurate [16–18] (Table 1).

In this case of PHNET, the initial tumor was diagnosed as a G2 tumor (mitotic count, 16 or 17 mitoses/10 HPFs; Ki-67 index, 20%); the recurrent tumor was a G1 tumor (mitotic count, 1 mitosis/10 HPFs; Ki-67 index, 2%) and the metastasis was G2 (mitotic count, 2 mitoses/10 HPFs; Ki-67 index, 5%). This variation of Ki-67 index within individual NETs or within their metastasis is common, and the higher Ki-67 index value decides the tumor grade [19]. Interestingly, metastatic tumors tend to mimic the histopathologic and immunohistochemical characteristics of the primary tumor in the liver [9], which was noted in this PHNET lung metastasis.

Another enigma surrounding PHNETs is how they behave in the long term. Our case showed both recurrence after a left hepatic lobectomy and metastasis to the lung during the 13-year course. A 7-year retrospective study by Li et al. [20] reviewed 10 PHNET cases, of which 4 cases (with both G2 and G3) had metastasis at presentation. Three of these 4 cases had metastasis to hepatic hilar lymph nodes and 1 case had metastasis to bone and skeletal muscle. Another retrospective study by Park et al. [1] reviewed 12 cases of G3 PHNETs, of which 3 had metastasis: 2 metastasized to bone and lymph node and 1 metastasized to supraclavicular lymph node. No case of distant metastasis to the lung was found in our literature review. In addition and similar to this case, PHNETs with a Ki-67 index as low as 5% [20] can metastasize, making both the diagnosis of the PHNETs and the prediction of their aggressiveness difficult.

Resection continues to be the mainstay of therapy for localized disease, with a 5-year survival rate of 78% to 80% and a recurrence risk of 18% to 26% [21]. In unresectable cases, transarterial chemoembolization and liver transplant are potential treatment options [1]. Furthermore, chemotherapeutic agents (i.e., 5-fluorouracil, doxorubicin, mitomycin, etoposide, and cisplatin) are an option for PHNETs with metastasis, but the benefit of chemotherapy is questionable [1]. Follow-up is important with PHNET because cases of recurrence have been documented as late as 10 to 13 years postresection [22, 23].

In conclusion, PHNETs are rare tumors but should be included in differential diagnoses of primary hepatic tumors, especially after other sites of origin have been excluded. PHNETs can present with varying histologic findings, but immunostaining for neuroendocrine markers is the gold standard for diagnosis. Independent of tumor grade, long-term follow-up is necessary after treatment because evidence shows late recurrence in the few follow-up case reports. PHNETs are poorly described, and most likely they are an underdiagnosed entity with the tumor prognosis largely unknown.

## Figures and Tables

**Table 1:** World health organization classification of gastroenteropancreatic neuroendocrine tumors.

Grade	Origin
Low	<2 mitoses/10 HPFs *and* <3% Ki-67 index
Intermediate	2–20 mitoses/10 HPFs *or* 3%–20% Ki-67 index
High	>20 mitoses/10 HPFs *or* >20% Ki-67 index

HPF, high-power field.

(Adapted from Klimstra et al. [16]).

## Abbreviations

G1: Low grade
G2: Intermediate grade
G3: High grade
HPF: High-power field
MRI: Magnetic resonance imaging
NET: Neuroendocrine tumor
PHNET: Primary hepatic neuroendocrine tumor
TTF1: Thyroid transcription factor.
